# Neurodevelopmental Challenges: Dealing with ADHD and Addictions

**DOI:** 10.1192/j.eurpsy.2025.200

**Published:** 2025-08-26

**Authors:** A. Schellekens

**Affiliations:** Psychiatry, Radboudumc, Nijmegen, Netherlands

## Abstract

**Abstract:**

Attention Deficit Hyperactivity Disorder (ADHD) and Substance Use Disorders (SUD) frequently co-occur. Patients with this comorbidity report poorer qualtiy of life, and have worse treatment outcomes for both disorders, including high relapse rates of the SUD. In this case based discussion, clinical dilemma’s in the diagnosis and treatment of patients with this comorbidity will be discussed. Recent findings of the International Collaboration on ADHD and Substance Abuse (ICASA) will be used as background, including findings from ICASA’s most recent work: the longitudinal International Naturalistic Cohort Study of ADHD and SUD (INCAS), where nearly 600 patients with both ADHD and SUD in treatment were followed for nine months. In addition, ICASA’s consensus statements on diagnosis and treatment of co-occurring ADHD and SUD in youths and adults will be shortly discussed.

**Disclosure of Interest:**

None Declared

